# Integrating haptic simulation in dentistry: faculty insights and future directions

**DOI:** 10.3389/froh.2025.1592095

**Published:** 2025-08-06

**Authors:** Danya Hashem, Abeer Farag, Amnah A. Algarni, Rania Zahran Mubarak, Nisreen Nabiel Hassan, Anfal Alqussier, Somaya Ali Saleh

**Affiliations:** ^1^ Department of Restorative Dental Science, College of Dentistry, Taibah University, Madinah, Saudi Arabia; ^2^ Department of Restorative Dentistry, Faculty of Dentistry, Minia University, Minia, Egypt; ^3^ Department of Operative Dentistry, Faculty of Dentistry, Ain Shams University, Cairo, Egypt

**Keywords:** haptic virtual reality simulation, simodont dental trainer, faculty perception, clinical training, dental education

## Abstract

**Purpose:**

Evaluate the knowledge of faculty members about haptic virtual reality simulation (HVRS) in dentistry and assess faculty members' perception towards the use of HVRS in dental education.

**Methods:**

This interventional study involved distributing a questionnaire to 29 faculty members from different dental specialties as a baseline before attending a hands-on workshop on HVRS followed by another questionnaire distributed after the workshop. Data was analyzed using IBM SPSS version 26, employing chi-squared tests for statistical significance.

**Results:**

Around 69% of faculty members had heard of HVRS prior to the study, but 86% had no prior experience using it. There was an increase in the willingness to use HVRS in teaching and to be included as an adjunct to pre-clinical and clinical training after attending the workshop. Post-training assessments indicated a significant shift in perceptions, with faculty expressing high satisfaction regarding the ergonomic design, visual system, and usability of the Simodont® simulator. Participants agreed that HVRS could enhance students' manual skills and self-evaluation capabilities.

**Conclusion:**

Faculty members at Taibah University expressed a positive perception of the use of HVRS in dental education and a willingness to adopt HVRS as a teaching aid. They intend to incorporate it into pre-clinical and clinical training. Although the study has a small sample size, it highlights the need for ongoing faculty training to facilitate the integration of HVRS technology in dental education, while also calling for further research to explore its long-term effects on learning outcomes.

## 1 Introduction

Training in dentistry focuses on the development of manual skills to achieve competence and provide safe and effective clinical dental care. Bridging the gap and reducing the difference between training in a pre-clinical artificial environment and the real clinical environment has been the focus of many studies over the years (1). The advent of haptic virtual reality simulation (HVRS) has brought about a significant advancement in digital technology, demonstrating its effectiveness as an educational tool. It offers sensory feedback for enamel and dentine preparation, allowing students to practice and refine their psychomotor skills as many times as needed, without adding to staff workload (2–5). Among the many devices available in the market is Simodont®, a pioneer specifically in the field of restorative dentistry (6–8). However, one barrier to the implementation of such technology is the lack of trained educators, which may affect their willingness to adopt and incorporate this technology as an adjunct to pre-clinical and clinical training in dentistry. Indeed, a study has found that although dental students, educators, and practitioners have a positive attitude towards haptic virtual reality-based technologies, very few have used it in education and practice (9). One reason for this is the lack of haptic virtual reality simulators in many teaching schools due to the extensive investment needed for initial outlay costs and ongoing funding allocations for maintenance and software updates and supervisory staff training (5, 10). Nevertheless, the use of HVRS is increasing in popularity as more studies have proved its efficiency and benefits when used in conjunction with conventional training (1, 4, 11, 12). Moreover, its importance has been emphasized following the novel coronavirus SARS-Co-V-2 (severe acute respiratory syndrome coronavirus 2) pandemic in late 2019 which called for social distancing. This demanded the replacement of traditional teaching methods with digital or virtual set-ups whenever possible (13). This has led to the application and adoption of this technology by many dental schools. Incorporating HVRS into undergraduate and continuing education programs could influence dental practitioners' willingness to adopt new technologies and improve their ability to critically assess emerging trends in dentistry (9). The knowledge, attitude and experience of teaching staff is considered a cornerstone for the adoption and implementation of HVRS in the educational process. The College of Dentistry at Taibah University in Madinah has invested on Simodont® simulators for use as an adjunct to pre-clinical and clinical training, educational research, and to facilitate a waste-free environment. This study aimed first to evaluate the knowledge of faculty members at Taibah University College of Dentistry in Madinah about HVRS in dentistry and to assess their perception towards the use of HVRS in dental education. Second, to explore whether the lack of knowledge or experience in using haptic simulation among faculty members could be considered as a potential barrier to its use in the educational process. The first null hypothesis is that faculty members at Taibah University College of Dentistry have no significant knowledge about HVRS in dentistry and no significant perception towards the use of HVRS in dental education. The second null hypothesis is that there is no association between faculty members' lack of knowledge or experience with HVRS and its use as a barrier in the educational process.

The study would help shed light on the current knowledge gaps among faculty regarding HVRS. Identifying these barriers is essential for developing targeted training programs that can facilitate the adoption of such technologies in dental education. Insights gained from faculty perceptions can inform curriculum designers and educational leaders about the feasibility and effectiveness of integrating HVRS into dental training, ultimately leading to more effective educational strategies.

## 2 Materials and methods

Ethical approval was granted from Taibah University College of Dentistry Research Ethics Committee (TUCDREC/230523/DHashem). An announcement was made for all dental faculty members interested in the topic of HVRS to attend a workshop on HVRS at the College of Dentistry in the campus as part of the continuous development workshops conducted in the College. On the day of the workshop, it was explained to all participants that a research study would be conducted along with the workshop and that participation was completely voluntary, and not agreeing to participate in the research study will not affect the participant in the workshop in any way. The purpose of the research study was then described and a baseline questionnaire in the form of a link (Google form) was distributed to all participating faculty members before starting the workshop which involved their knowledge and perception towards the use of HVRS in dental education. Written informed consent was obtained from all the faculty members who agreed to participate in the study as part of the questionnaire and they were ensured that the data will be kept confidential. Following that, a lecture lasting for two hours was given to the participants by two professional well trained restorative staff members (AF) and (DH). The lecture contained information about the different HVRS with a focus on the Simodont dental trainer (Moog Industrial Group, Nieuw-Vennep, Netherlands) which is equipped with courseware software developed by the Academic Centre for Dentistry Amsterdam (ACTA, Amsterdam, Netherlands). The lecture also involved operation of the Simodont® dental trainer, orientation by all the virtual tools and instruments and explanation of the manual dexterity exercises blocks and cariology case scenarios available in the Simodont® dental trainer courseware software.

**Figure 1:**
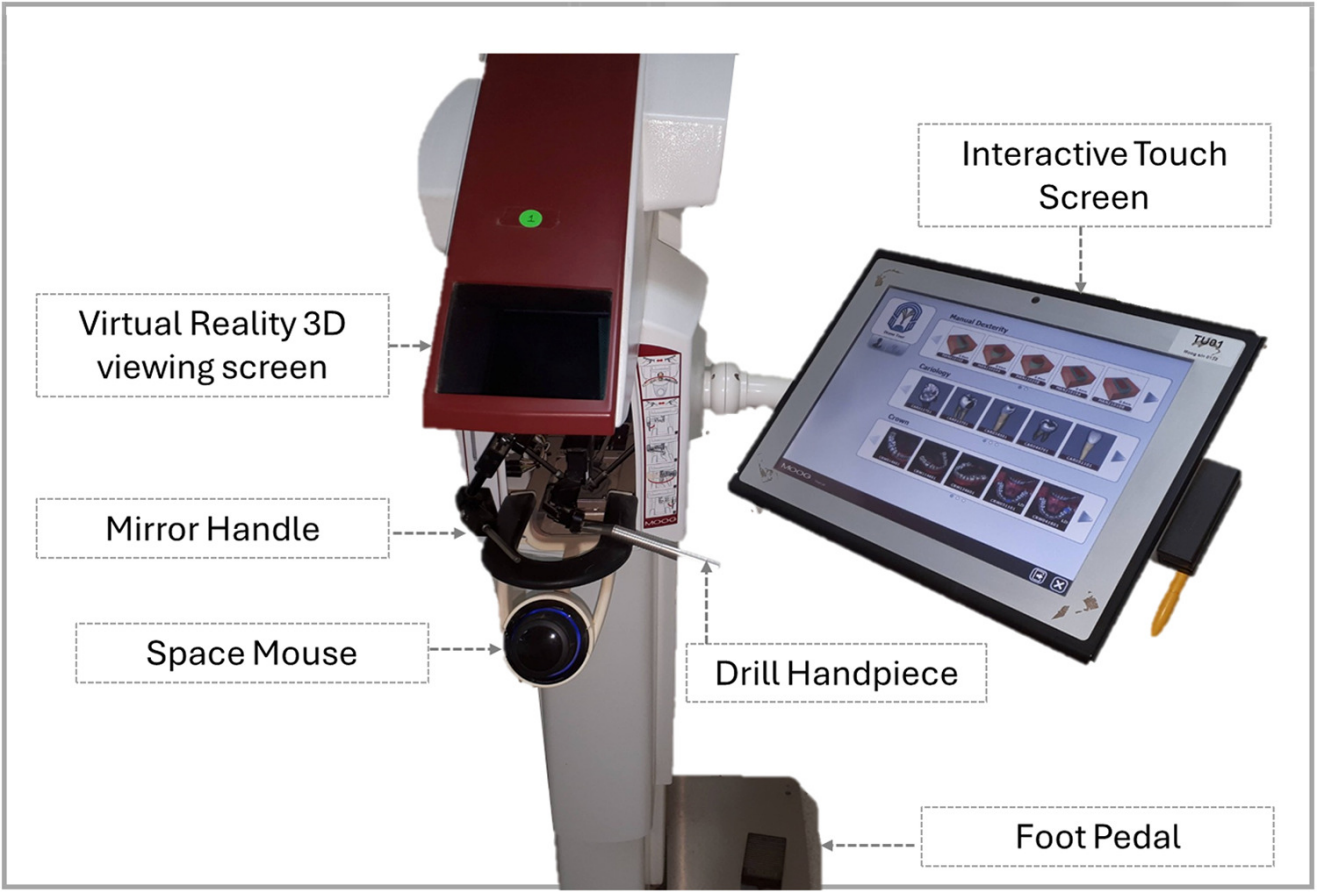
HVRS simodont® trainer and its components.

Then, hands-on training on the use of HVRS was given to all participants in the HVRS lab for five hours. The course software includes different options of manual dexterity exercises blocks, virtual sound and carious teeth for operative procedures, and different virtual hand and rotary instruments including a choice for right or left-handed users. All the participants used six Simodont® dental trainers available in the HVRS lab. Each participant trained for 50–60 minutes on drilling the manual dexterity blocks and the different cariology cases available in the software using virtual instruments and drills implemented in the simulator's software (Figure 1).

At the end of the workshop, another online questionnaire was distributed to all consented participants. This post-workshop questionnaire assessed the faculty members' perception towards the use of HVRS in dental education after gaining information and training about it.

The questionnaire was adapted from Gal et al. and de Boer et al. (14, 15) and was tested for validity and reliability on a sample of faculty members. The questionnaire comprised demographics and general questions on HVRS, the extent of agreement to statements regarding ergonomics, usability, reality, and staff opinions on HVRS, and a section with a Likert scale from 0 to 5 on several statements about HVRS and finally, three open-ended questions involving the best time to introduce Simodont training to students, advantages and disadvantages of the use of Simodont®.

### 2.1 Statistical analysis

The data in this study is presented as frequencies and percentages. Statistical analysis was performed using IBM SPSS version 26. The chi-squared test was used to compare the results before and after the session for all comparisons. A *p*-value less than or equal to 0.05 was considered statistically significant. Qualitative analysis was conducted to identify themes in the open-ended responses. The thematic analysis was carried out using a staged approach.

## 3 Results

### 3.1 Participants

A total of 31 faculty members attended the workshop and 29 faculty members from various dental specialties participated in the study, including dental education (*n* = 2), oral and maxillofacial surgery (*n* = 5), oral basic and clinical sciences (*n* = 1), pediatric dentistry (*n* = 2), preventive dental sciences (*n* = 4), restorative dental sciences (*n* = 9), and substitutive dental sciences (*n* = 6). There were 4 professors, 8 associate professors, 16 assistant professors, and 1 lecturer. The participant population was 72.4% female and 27.6% male.

### 3.2 Prior experience and willingness to use HVRS

Table 1 demonstrates that most participants (69%) had heard of HVRS before the study, but most had no experience using it (86%). Only one participant used HVRS for teaching. Before using the simulator, 41.4% of participants were willing to use HVRS in teaching, compared to 55.2% after using the simulator. However, this difference was not statistically significant.

**Table 1:** Prior experience with HVRS before and after the training session.

Prior experience with HVRS		Pre-questionnaire frequency (%)	Post-questionnaire frequency (%)	*P*-value
Have you heard about Haptic Virtual Reality Simulation (HVRS)?	No	9 (31)	3 (10.3)	0.052
	Yes	20 (69)	26 (89.7)	
Do you have any previous experience with using HVRS?	No	25 (86.2)	13 (44.8)	<0.001
	Yes	4 (13.8)	16 (55.2)	
If yes, how would you rate your experience?	1 (Low)	14 (48.3)	13 (44.8)	0.201
	2	3 (10.3)	8 (27.6)	
	3 (High)	0 (0)	1 (3.4)	
Have you used HVRS in teaching?	No	28 (96.6)	28 (96.6)	1
	Yes	1 (3.4)	1 (3.4)	
Would you use HVRS in teaching?	Maybe	11 (37.9)	11 (37.9)	0.276
	No	6 (20.7)	2 (6.9)	
	Yes	12 (41.4)	16 (55.2)	
Do you think HVRS should be included as an adjunct to pre-clinical and clinical training?	Maybe	5 (17.2)	3 (10.3)	0.243
	No	2 (6.9)	0	
	Yes	22 (75.9)	26 (89.7)	

### 3.3 Opinion on HVRS as an adjunct to pre-clinical and clinical training

Before using the simulator, 75.9% of participants thought that HVRS should be included as an adjunct to pre-clinical and clinical training. This proportion increased to 89.7% after using the simulator. However, this difference was also not statistically significant (Table 1).

### 3.4 Ergonomics

After the training session, there was a significant increase in the proportion of participants who agreed that Simodont® simulates a practitioner working in an upright position (82.8% compared to 41.1% before the training session), that the wrist support during drilling avoids fatigue (69% compared to 27.6%), and that the visual system is clear enough (86.2% compared to 27.6%). However, 10% of practitioners suffered from disorientation, headache, and nausea after the session (Table 2).

**Table 2:** Participants’ opinions on ergonomics of HVRS before and after the training session.

Ergonomics		Pre-questionnaire frequency (%)	Post-questionnaire frequency (%)	*P*-value
The Simodont® simulator stimulates you to work in an upright position	Agree	12 (41.1)	24 (82.8)	<0.001
	Neutral	0	5 (17.2)	
	Disagree	1 (3.4)	0	
	I don't know	16 (55.2)	0	
The wrist support during drilling avoids hand fatigue	Agree	8 (27.6)	20 (69)	<0.001
	Neutral	0	8 (27.6)	
	Disagree	2 (6.9)	1 (3.4)	
	I don't know	19 (65.5)	0	
The visual system is clear enough	Agree	8 (27.6)	25 (86.2)	<0.001
	Neutral	0	4 (13.8)	
	Disagree	3 (10.3)	0	
	I don't know	18 (62.1)	0	
I suffered from disorientation, headache, nausea, after using the Simodont® simulator	Agree	0	3 (10.3)	<0.001
	Neutral	0	6 (20.7)	
	Disagree	5 (17.2)	20 (69.0)	
	I don't know	23 (79.3)	0	

### 3.5 Usability and reality

Table 3 shows that after using the simulator, there was a statistically significant increase in the proportion of participants who agreed that the simulator instructions are clear (96.6% compared to 10.3%), the courseware is easy to manipulate/approach (93.1% compared to 6.9%), Simodont cannot replace the teacher (96.6% compared to 24.1%), and the teacher should give further feedback (96.6% compared to 24.1%). Table 4 shows that after using the simulator, there was a statistically significant increase in the proportion of participants who agreed that the simulator provides a realistic experience in teeth preparation.

**Table 3:** Participants’ opinions on the usability of HVRS before and after the training session.

Usability		Pre-questionnaire frequency (%)	Post-questionnaire frequency (%)	*P*-value
Simodont® simulator instructions are clear	Agree	3 (10.3)	28 (96.6)	<0.001
	Neutral	0	1 (3.4)	
	Disagree	5 (17.2)	0	
	I don't know	21 (72.4)	0	
The courseware is easy to manipulate/approach	Agree	2 (6.9)	27 (93.1)	<0.001
	Neutral	0	2 (6.9)	
	Disagree	4 (13.8)	0	
	I don't know	23 (79.3)	0	
Simodont® cannot replace the teacher	Agree	7 (24.1)	28 (96.6)	<0.001
	Neutral	0	1 (3.4)	
	Disagree	5 (17.2)	0	
	I don't know	17 (58.6)	0	
Teacher should give further feedback	Agree	7 (24.1)	28 (96.6)	<0.001
	Neutral	0	1 (3.4)	
	Disagree	5 (17.2)	0	
	I don't know	17 (58.8)	0	

**Table 4:** Participants’ opinions on the reality of HVRS before and after the training session.

Reality		Pre-questionnaire frequency (%)	Post-questionnaire frequency (%)	*P*-value
Drilling the manual dexterity box is similar to drilling in plastic teeth]	Agree	0	13 (44.8)	<0.001
	Neutral	0	9 (31)	
	Disagree	5 (17.2)	7 (24.1)	
	I don't know	24 (82.8)	0	
Hardness of enamel simulates natural enamel	Agree	3 (10.3)	13 (44.8)	<0.001
	Neutral	0	10 (34.5)	
	Disagree	2 (6.9)	6 (20.7)	
	I don't know	24 (82.8)	0	
Hardness of dentine simulates natural dentine	Agree	4 (13.8)	14 (48.3)	<0.001
	Neutral	0	10 (34.5)	
	Disagree	2 (6.9)	5 (17.2)	
	I don't know	23 (79.3)	0	
The hardness of enamel is easily distinguished from the hardness of dentine	Agree	1 (3.4)	13 (44.8)	<0.001
	Neutral	0	12 (41.4)	
	Disagree	3 (10.3	4 (13.8)	
	I don't know	25 (86.2)	0	
The mirror image are true to size and form	Agree	1 (3.4)	16 (55.2)	<0.001
	Neutral	0	6 (20.7)	
	Disagree	3 (10.3)	7 (24.1)	
	I don't know	25 (86.2)	0	
The dental hand mirror allows a realistic inspection of teeth from all aspects	Agree	2 (6.9)	16 (55.2)	<0.001
	Neutral	0	8 (27.6)	
	Disagree	4 (13.8)	5 (17.2)	
	I don't know	23 (79.3)	0	
The image in the Visual display is true in size and form to the visual scene	Agree	2 (6.9)	19 (65.5)	<0.001
	Neutral	0	9 (31)	
	Disagree	2 (6.9)	1 (3.4)	
	I don't know	25 (86.2)	0	
Sound of the air rotor is similar to actual one	Agree	2 (6.9)	18 (62.1)	<0.001
	Neutral	0	9 (31)	
	Disagree	3 (10.3)	2 (6.9)	
	I don't know			
Sound of the low speed motor is similar to actual motor	Agree	2 (6.9)	16 (55.2)	<0.001
	Neutral	0	13 (44.8)	
	Disagree	3 (10.3)	0	
	I don't know	24 (82.8)	0	
The drill speed under the control of foot pedal is realistic	Agree	2 (6.9)	24 (82.8)	<0.001
	Neutral	0	4 (13.8)	
	Disagree	3 (10.3)	1 (3.4)	
	I don't know	24 (82.8)	0	
The drill speed and force exerted by the operator is realistic	Agree	2 (6.9)	21 (72.4)	<0.001
	Neutral	0	6 (20.7)	
	Disagree	3 (10.3)	2 (6.9)	
	I don't know	24 (82.8)	0	
Vibration of the drill hand piece is transmitted to the operator hand	Agree	3 (10.3)	22 (75.9)	<0.001
	Neutral	0	7 (24.1)	
	Disagree	2 (6.9)	0	
	I don't know	24 (82.8)	0	
Rotary Instrument type, shape and size are suitable	Agree	3 (10.3)	25 (86.2)	<0.001
	Neutral	0	4 (13.8)	
	Disagree	2 (6.9)	0	
	I don't know	24 (82.8)	0	
Hand instruments types, size and shape are suitable	Agree	3 (10.3)	25 (86.2)	<0.001
	Neutral	0	3 (10.3)	
	Disagree	3 (10.3)	1 (3.4)	
	I don't know	23 (79.3)	0	
Simodont® simulator provides a realistic sense of touch in virtual environment	Agree	5 (17.2)	21 (72.4)	<0.001
	Neutral	0	8 (27.6)	
	Disagree	2 (6.9)	0	
	I don't know	22 (75.9)	0	

### 3.6 Staff opinion

According to Table 5, there was a statistically significant increase in the proportion of staff members who have a positive opinion on the Simodont® simulator after using it. The chi-square test results in Table 6 demonstrate that there was a statistically significant difference in the proportion of participants who gave a rating of 4 or 5 on each of the seven questions about the Simodont simulator before and after using it.

**Table 5:** Participants’ general opinions on HVRS before and after the training session.

Staff opinion		Pre-questionnaire frequency (%)	Post-questionnaire frequency (%)	*P*-value
The simulator reproduced teeth drilling in a realistic way.	Agree	4 (13.8)	21 (72.4)	<0.001
	Neutral	0	7 (24.1)	
	Disagree	3 (10.3)	1 (3.4)	
	I don't know	22 (75.9)	0	
The student skills in teeth drilling will improve after practice with Simodont	Agree	5 (17.2)	24 (82.8)	<0.001
	Neutral	0	5 (17.2)	
	Disagree	3 (10.3)	0	
	I don't know	21 (72.4)	0	
The practice in Simodont® simulator will provide the student with new knowledge	Agree	4 (13.8)	26 (89.7)	<0.001
	Neutral	0	2 (6.9)	
	Disagree	3 (10.3)	1 (3.4)	
	I don't know	22 (75.9)	0	
Practice in the simulator should be included in the course in the future	Agree	5 (17.2)	26 (89.7)	<0.001
	Neutral	0	3 (10.3)	
	Disagree	3 (10.3)	0	
	I don't know	21 (72.4)	0	
I would recommend more training with the simulator	Agree	5 (17.2)	28 (96.6)	<0.001
	Neutral	0	1 (3.4)	
	Disagree	3 (10.3)	0	
	I don't know	21 (72.4)	0	
The patient cases in Simodont® will give the student more knowledge about the variation and difficulties of the different operative cases	Agree	4 (13.8)	25 (86.2)	<0.001
	Neutral	0	3 (10.3)	
	Disagree	3 (10.3)	1 (3.4)	
	I don't know	22 (75.6)	0	
Simodont® will give the student more knowledge of whether a case should be referred or not	Agree	3 (10.3)	22 (75.9)	<0.001
	Neutral	0	5 (17.2)	
	Disagree	3 (10.3)	2 (6.9)	
	I don't know	22 (75.9)	0	
I would recommend having more Simodont cases to work with	Agree	4 (13.8)	26 (89.7)	<0.001
	Neutral	0	2 (6.9)	
	disagree	3 (10.3)	1 (3.4)	
	I don't know	22 (75.9)	0	
Simodont® will improve the student's knowledge regarding diagnostics operative dentistry	Agree	4 (13.8)	26 (89.7)	<0.001
	Neutral	0	2 (6.9)	
	Disagree	4 (13.8)	1 (3.4)	
	I don't know	21 (72.4)	0	

**Table 6:** Participants’ answers to questions about HVRS before and after the training session on a Likert scale from 1 to 5.

Question	Likert scale	Pre-questionnaire frequency (%)	Post-questionnaire frequency (%)	*P*-value
To what extent can the simulator be helpful in teaching manual skills in dentistry?	1^a^	3 (10.3)	0	0.063
	2	1 (3.4)	0	
	3	11 (37.9)	5 (17.2)	
	4	7 (24.1)	13 (44.8)	
	5	7 (24.1)	11 (37.9)	
To what extent can the simulator be useful in self-training of manual skills in dentistry?	1	2 (6.9	0	0.017
	2	1 (3.4)	0	
	3	12 (41.4)	3 (10.3)	
	4	7 (24.1)	13 (44.8)	
	5	7 (24.1)	13 (44.8)	
To what extent can the simulator be useful in evaluating manual skills in dentistry?	1	3 (10.3)	0	0.117
	2	2 (6.9)	1 (3.4)	
	3	13 (44.8)	8 (27.6)	
	4	6 (20.7)	9 (31)	
	5	5 (17.2)	11 (37.9)	
To what extent is the sensation provided by the simulator in cariology cases similar to drilling in a real tooth?	1	4 (13.8)	0	0.018
	2	3 (10.3)	0	
	3	13 (44.8)	12 (41.4)	
	4	4 (13.8)	13 (44.8)	
	5	5 (17.2)	4 (13.8)	
To what extent is the sensation provided by the simulator in exercises blocks similar to drilling in an acrylic tooth?	1	3 (10.3)	1 (3.4)	0.029
	2	2 (6.9)	3 (10.3)	
	3	18 (62.1)	10 (34.5)	
	4	2 (6.9)	12 (41.4)	
	5	4 (13.8)	3 (10.3)	
To what extent is the grip of the simulator hand piece similar to a high-speed turbine grip?	1	4 (13.8)	0	0.013
	2	2 (6.9)	0	
	3	14 (48.3)	8 (27.6)	
	4	5 (17.2)	14 (48.3)	
	5	4 (13.8)	7 (24.1)	
To what extent is the use of the simulator comfortable?	1	4 (13.8)	0	0.011
	2	2 (6.9)	1 (3.4)	
	3	14 (48.3)	6 (20.7)	
	4	5 (17.2)	14 (48.3)	
	5	4 (13.8)	8 (27.6)	

^a^ Where 1 is the lowest score and 5 is the highest score.

### 3.7 Thematic analysis of responses to open ended questions

Participants were asked about the best timing to introduce Simodont® training during the pre-clinical and clinical dentistry courses. The most common recommendation provided by participants before they used the simulator was to introduce Simodont® training at the beginning of the preclinical course (44.8%). After using the simulator, the recommendation was to introduce Simodont training during the preclinical courses (37.9%), before preclinical courses (3.4%), at the beginning of preclinical course (3.4%) and as workshops during summer (3.4%).

When asked about the main advantages of the Simodont®, the answers provided by the participants revolved around three main themes: The first theme involves safety and reduced risk in a controlled learning environment**.** Specific examples include: “It allows students to make mistakes without any harm to real patients, which is essential in early training”. Another example includes: “Simodont® eliminates the ethical and safety concerns of practicing on live patients during the initial skill-building phase”. And also, “Students can repeatedly practice procedures until they gain confidence, without endangering patient well-being”.

The second theme revolves around: Improving learning and skill development through enhancing cognitive and psychomotor abilities essential for dentistry. Examples include: “The tactile feedback helps students differentiate between enamel and dentin, which sharpens diagnostic and manual dexterity skills”. Another example: “It facilitates independent learning; students can track their own progress and correct errors on the spot”. “Students gain a sense of achievement and confidence before transitioning to real patients”. “It encourages critical thinking and decision-making, especially when combined with virtual case scenarios”.

The third theme revolved around other benefits focusing on resource efficiency and realism in training such as: “We’ve saved significantly on consumables like plastic teeth and typodonts since integrating Simodont® into our preclinical labs”. “It reduces the teacher's burden; instructors can focus on giving feedback rather than demonstrating the same steps repeatedly”. “The 3D realism of the simulation mimics the feel of actual tooth structures better than traditional models”. “It shortens the learning curve when students move to clinical practice, as they’re already familiar with the tactile experience”.

When asked about the main disadvantages of Simodont, three categories emerged: Firstly, cost of initial setup, frequent need of updating the system, the need for highly trained maintenance personnel, and staff members training. Secondly, being less worthy for students' training in clinical settings due to lack of patient interaction and does not mimic the real oral environment. Other miscellaneous disadvantages mentioned were: It caused a headache after being used for a long period of time, the need for specially trained staff. They also mentioned that it is not sufficient for training by itself but must be used as an adjunct to conventional and phantom head training methods.

## 4 Discussion

Incorporating HVRS technology into educational settings requires assessing several factors that affect acceptance of or resistance to its integration. These factors include faculty members' perceptions, barriers to implementation, incentives for adoption, previous experience with technology, students' perceptions, and institutional support (16). Investigating these aspects can help ensure the effective utilization of HVRS technology in academic environments (17). The Simodont dental trainer used in this study is a haptic virtual reality simulator (HVRS), which offers an additional dimension to virtual reality through sense of touch of preparation on enamel and dentine through a robotic force feedback arm which is known as the haptic effect. In this study the faculty members received knowledge and practice on HVRS Simodont and developed their own opinion upon real experience. This was obviously clear from the scale of (I don't know) in the pre training questionnaire which changes to Agree, Neutral and rarely disagree in all post training questionnaire items.

Before training, most of the faculty members had heard of HVRS and were willing to use it in teaching. They thought that HVRS should be included as an adjunct to pre-clinical and clinical training. After training on HVRS the faculty members were highly satisfied with the ergonomic, visual system, reality, and useability of the simulator. The faculty members' opinion that HVRS will enhance the students' manual skills, self-training, and self-evaluation agreed with several previous studies (2, 4, 7, 15). This also is consistent, to some extent, with another study where participants expected the HVRS to be beneficial in teaching manual skills, self-learning of manual skills, and to a lesser degree, the evaluation of manual skills (14). Meanwhile Vincent et al., reported no significant difference in psychomotor skills acquisition (quality and time of cavity preparation) using Virteasy HVRS and conventional cavity preparation using plastic teeth; however, they found it suitable in saving the teacher's time (18).

The faculty members in this study agreed that teachers should give further feedback to the students in addition to the educational feedback provided by the Simodont®. This was in agreement with another study that emphasized staff member instructions to be crucial in any educational process (19). Another study found better acquisition of psychomotor skills in novice students through a combination of instructor and HVRS Simodont® feedback, compared to the HVRS Simodont® feedback or instructor's feedback alone (2).

The main advantages reported by faculty members regarding HVRS (Simodont®): a safe learning environment, improvement of psychomotor skills, saving time for both students and teaching staff. These were consistent with several previous reports stating that HVRS in restorative dentistry are a promising pedagogical tool for training novice students. It allows for a faster acquisition and improvement of psychomotor skills, better cavity preparation quality, reduced working time, improve student ability for self-evaluation (2, 4, 7, 15, 20, 21). Also, Nassar and Tekian (19), emphasized the benefits of the simulation in the field of operative dentistry, especially in saving faculty time as well as allowing the students to perform repeated attempts to achieve mastery at their own pace. However, they recommended further investigations in a well-controlled trial and long-term follow-up. Another study emphasized that VR/HVRS is a useful adjunct to conventional learning in dentistry. However, further trials are required as well as standardization and accreditation (21). Indeed, HVRS can enhance dental education leading to better-prepared graduates. This improved training translates to higher-quality patient care, with reduced errors and more confident practitioners.

Regarding the disadvantages of Simodont HVRS, faculty members pointed out the cost of the initial set up of the system, maintenance, and updates which were consistent with other studies (22–24). Indeed, the initial investment in HVRS can be substantial, encompassing expenses for hardware, software, and specialized equipment. Moreover, to maintain the efficacy and relevance of these systems, continuous updates are required to incorporate advancements in dental procedures and to ensure compatibility with evolving technological standards. This necessitates a dedicated budget for software upgrades, hardware calibration, and technical support. Additionally, the rapid pace of technological innovation means that simulators can quickly become outdated, further emphasizing the need for regular updates to keep pace with new developments in dental education and practice (22–24).

Lack of patient interaction and no simulation of the real oral environment were other disadvantages that were mentioned. These observations previously prompted Yamaguchi et al. to create a virtual patient face model, which demonstrated significant promise in helping trainees consider patients' emotional responses, such as expressions of pain and anxiety, within virtual reality settings (3). Furthermore, two other studies proposed that artificial intelligence can be employed to simulate lifelike conversation, providing an even more “realistic” simulation of real clinical practice (25, 26). A few faculty members also mentioned that Simodont® HVRS caused headaches after being used for a long period of time. This was mentioned in a previous study that reported discomfort and motion sickness of some users during prolonged use of the VR headset (27). Finally, faculty members pointed out that Simodont® HVRS must be used as an adjunct to conventional and phantom head training methods. This was in agreement with many other studies (2, 4, 7, 15, 19, 21, 24, 28). The use of mixed reality (MR) technology whereby the virtual component of MR can be utilized to create a simulated oral environment that replicates hard and soft oral tissues as well as dental instruments, helping to reduce material use during skills training. A physical phantom head can represent the patient's head in the real world and be aligned with the virtual environment, addressing challenges related to patient positioning in clinical scenarios. When combined with haptic feedback devices, this setup enables the development of a dental tooth preparation simulator that closely mimics actual clinical conditions, offering users a more immersive and realistic training experience (29, 30).

This study has its limitations which include a relatively small sample of 29 faculty members, which may not fully represent the broader faculty population across different dental schools potentially affecting the generalizability of the findings. Further studies are needed to include a larger and more diverse group of faculty members from various dental institutions to enhance the generalizability of the findings. Research should be conducted over a longer period to evaluate the sustained impact of HVRS on both faculty teaching practices and student learning outcomes. Additionally, future research should investigate the specific challenges faculty could encounter when integrating HVRS into their curricula, as well as strategies to overcome these barriers. These recommendations aim to build on the findings of the current study and further explore the role of HVRS in dental education.

## 5 Conclusion

Faculty members have a positive perception regarding using HVRS in dental education. The findings indicate that after training with the Simodont® dental trainer, there was a significant increase in agreement among participants regarding the usability and effectiveness of the simulator in providing realistic training experience. Faculty members recognized the potential of HVRS to enhance students' manual dexterity and knowledge in operative dentistry, suggesting that it could be a valuable tool in dental education. Additionally, the study highlights the importance of further training and integration of such technologies into the curriculum to maximize their benefits for both faculty and students.

## Data Availability

The original contributions presented in the study are included in the article/Supplementary Material, further inquiries can be directed to the corresponding author.
